# Linking Climate Trends to Population Dynamics in the Baltic Ringed Seal: Impacts of Historical and Future Winter Temperatures

**DOI:** 10.1007/s13280-012-0334-x

**Published:** 2012-08-01

**Authors:** Lisa Sundqvist, Tero Harkonen, Carl Johan Svensson, Karin C. Harding

**Affiliations:** 1 Department of Biological and Environmental Sciences, University of Gothenburg, P.O. Box 461, 405 30 Gothenburg, Sweden; 2 Swedish Museum of Natural History, P.O. Box 50007, 104 05 Stockholm, Sweden

**Keywords:** Global warming, Habitat loss, Ice coverage, Marine mammals, *Phoca hispida*, Pup survival

## Abstract

A global trend of a warming climate may seriously affect species dependent on sea ice. We investigated the impact of climate on the Baltic ringed seals (*Phoca hispida botnica*), using historical and future climatological time series. Availability of suitable breeding ice is known to affect pup survival. We used detailed information on how winter temperatures affect the extent of breeding ice and a climatological model (RCA3) to project the expected effects on the Baltic ringed seal population. The population comprises of three sub-populations, and our simulations suggest that all of them will experience severely hampered growth rates during the coming 90 years. The projected 30 730 seals at the end of the twenty-first century constitutes only 16 % of the historical population size, and thus reduced ice cover alone will severely limit their growth rate. This adds burden to a species already haunted by other anthropogenic impacts.

## Introduction

Global warming is causing major perturbations in ecosystems with extensive seasonal sea ice (Grebmeier et al. 2006). The potential of species to cope with ecosystem changes depends on their life histories and dispersal capacities. Pagophilic (“ice-loving”) seal species use sea ice for haul-out and breeding (Laidre et al. 2008; Siniff et al. 2008) as ice confers particular advantages compared to land, for instance protection against many predators and lower exposure to pathogens and parasites (Fay 1974; Jüssi et al. 2008). The timing of life-history events such as breeding, molt, and migration has evolved to match the annual changes in oceanographic conditions, where sea ice structure is one prominent factor (Moore and Huntington 2008). Seals breeding on ice are therefore exceptionally vulnerable not only to changes in the extent but also to changes in the distribution, and condition of seasonal sea ice (Tynan and DeMaster 1997; Jüssi et al. 2008; Laidre et al. 2008). While Arctic and Antarctic seal species have the possibility to migrate to higher latitudes, landlocked and semi-landlocked seal species such as the Baltic ringed seal (*Phoca hispida botnica*), the Caspian seal (*P. caspica*), the Baikal seal (*P. sibirica*), the Ladoga seal (*P. hispida ladogensis*), and the Saimaa seal (*P. hispida saimensis*) do not have this option. For these species reduced breeding habitats may pose an important density-dependent regulatory mechanism.

The Baltic ringed seal has been isolated from the Arctic ringed seal population since early postglacial times (Härkönen et al. 2005). The ringed seal population in the Baltic Sea amounted to about 190 000 seals in the beginning of the twentieth century, but intensive hunting and sterility due to organochlorine pollution caused a population crash, and only about 5000 seals remained in the late 1970s (Harding and Harkonen 1999). After hunting was prohibited and environmental conditions improved the population began to increase (Härkönen et al. 1998). The Baltic ringed seal inhabits three distinct areas in the Baltic Sea, the Bothnian Bay in the north, and the Gulf of Finland and the Gulf of Riga in the south (Härkönen et al. 1998).

Even for moderate scenarios of future warming sea ice in the Baltic Sea is expected to substantially reduce by the end of the twenty-first century (Meier et al. 2004), and this is likely to affect the ringed seals in several ways. Baltic ringed seals give birth to a single pup in the coldest winter months (February to early March) (Helle 1980). Females dig subnivean lairs offering protection against wind chill and predators (Lydersen and Gjertz 1986), and the pup is not likely to survive without this shelter (Helle 1980; Reeves 1998). The highest densities of ringed seals in the Baltic are found in compact and very close pack ice. This ice-type is the crucial breeding ice as snow accumulates here, which facilitates the construction of stable lairs (Härkönen et al. 1998). The seal pups depend on ice and snow cover throughout the lactation period, which last for a few months (Helle 1980; Laidre et al. 2008). Consequently, pups will not survive if breeding ice is not available, during warm winters no or almost no pup survival has been observed in the two southern Baltic ringed seal populations (Mart Jüssi, Pers. comm.). Adult ringed seals have small body sizes and the females have to feed during the lactation period (Lydersen and Kovacs 1999). Decreasing breeding ice will result in increasingly overlapping feeding habitats of the females as the density of seals on the ice increases. Consequently, diminishing ice fields initiate a density-dependent process affecting the weaning weight and survival rate of pups. We investigated if the extent of breeding ice in the Baltic Sea has been a limiting factor for historical population sizes, and model population effects for a future climatological scenario.

## Materials and Methods

### Aerial Surveys

Ringed seals in the Bothnian Bay were surveyed annually over the period 1988–2011 during peak molting time in the last week of April. All visible seals were counted within 800-m-wide strips, which were evenly positioned to cover 13 % of the total ice area. The strips were run alternatively from the northern fast ice to open water in the south and from south to north (Härkönen et al. 1998). Similar surveys were carried out in the Gulf of Finland 1992–1996 and in the Gulf of Riga in 1994 and 1996 (Härkönen et al. 1998), whereas later planned surveys in these areas were not performed due to deteriorating ice conditions and early break up of ice. Population sizes and growth rates were estimated from numbers of observed seals, where the visible proportion of the population was set at 60 %, which is the estimated minimum proportion hauled out during molt (Härkönen et al. 1998).

### Ringed Seal Breeding Ice and Future Temperatures

**Fig. 1:**
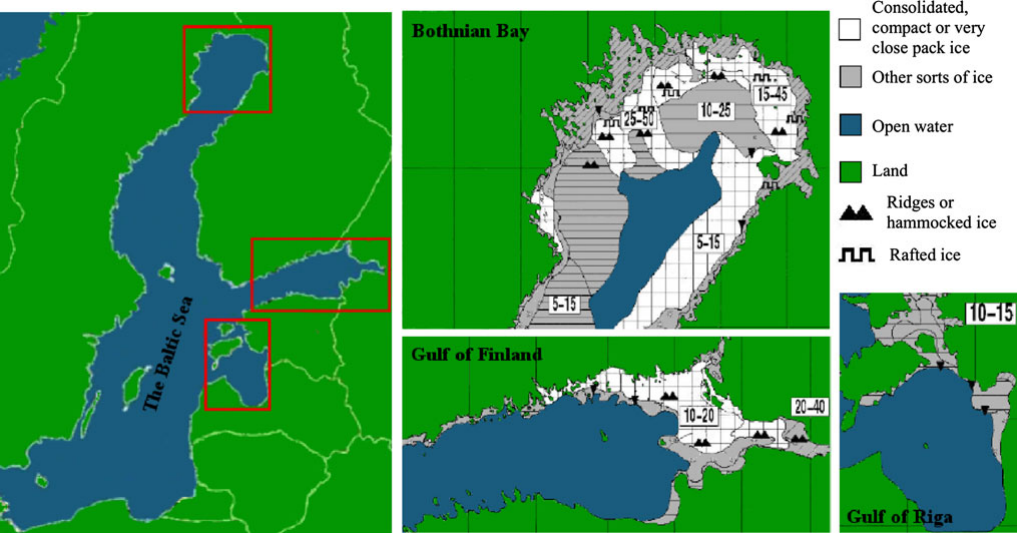
Regions in the Baltic Sea inhabited by ringed seals (*left*). Selected parts of a re-drawn ice-chart from Feb 10, 2009, where ice types are highlighted. The Bothnian Bay north of 64°N (*middle*, *top*), the Gulf of Finland east of 24°E (*middle*, *bottom*), and the Gulf of Riga south of 59°N and east of 22°E (*right*). The *square* marked/white ice (consolidated, compact or very close pack ice) is suitable ringed seal breeding ice

Baltic ringed seals require consolidated, compact, or very close pack ice for successful breeding (Fig. 1). The amount of appropriate breeding ice in early February will influence the pup survival that year. Detailed ice-charts are produced by the Swedish Meteorological and Hydrological Institute SMHI as a service to the cargo vessel traffic (Fig. 1), and based on these charts we measured the surface areas of suitable breeding ice (around 10th February each year) and correlated this to January mean air temperatures for each year for the period 1969–2011. Temperature data from Luleå airport (Sweden), provided by SMHI, were used for the Bothnian Bay (BB). Temperature measurements from Kotka Rankki (Finland) were provided by the Finnish Metrological Institute, and used for the Gulf of Finland (GoF) and the Gulf of Riga (GoR). Estimates of the extent of breeding ice were carried out by image analysis (ImageJ). Correlations between the areas of appropriate ringed seal breeding ice (BI_BB/GoF/GoR_) and January mean temperatures (*T*_BB/GoF/GoR_) for each region (Eqs. 1–3) were given by linear regressions, using the method of least squares.

1 $$ {\text{BI}}_{\text{BB}} = - 801T_{\text{BB}} + 3329\quad R^{2} = 0.38,\quad p < 0.001 $$

2 $$ {\text{BI}}_{\text{GoF}} = - 565T_{\text{GoF}} + 2573\quad R^{2} = 0.32,\quad p < 0.001 $$

3 $$ {\text{BI}}_{\text{GoR}} = - 523T_{\text{GoR}} - 865\quad R^{2} = 0.50,\quad p < 0.001 $$

**Fig. 2:**
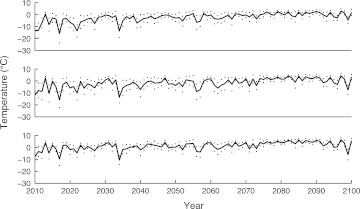
January average sea surface air temperatures (°C) with standard deviations for years 2010–2100, as given by the regional (RCA3) climatological model. The Bothnian Bay (*top*), the Gulf of Finland (*middle*), and the Gulf of Riga (*bottom*)

Future extents of ringed seal breeding ice for each region were projected using Eqs. 1–3 and a regional climatological model developed for the Baltic Sea area by the SMHI (Meier 2002; Kjellström et al. 2005). We used data from the Rossby Centre regional atmospheric climate model (RCA3) (Kjellström et al. 2005) to generate predictions for future average temperatures in January for the twenty-first century in three Baltic regions: the Bothnian Bay, the Gulf of Finland, and the Gulf of Riga (Fig. 2). The selected RCA3-scenario A1B_1 (Kjellström et al. 2005) is driven by a global climate model ECHAM5 (Roeckner 2011). We sub-sampled a set of 1000 trajectories in the time series 2010–2100 from the predicted January mean temperatures and standard deviations given by the RCA3 model. Through these trajectories and Eqs. 1–3, we estimate possible future areas of ringed seal breeding ice.

### The Demographic Model

**Fig. 3:**
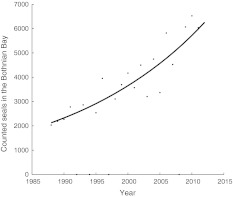
Population counts during aerial surveys 1988–2011 in the Bothnian Bay. Equation of trend line is $N_{t} = N_{0} \,*\,1.046^{t} ,\;R^{2} = 0.83$

Ringed seal life-history data were used to parameterize a Leslie matrix *A* (Leslie 1945). The dominant eigenvalue of the matrix is equivalent to the deterministic long-term annual growth rate (λ) (Caswell 2001). The Baltic ringed seal population growth rate is estimated to 1.046 from the aerial survey data (Fig. 3).

Ringed seals rear, at the most, one pup a year since year skipping is frequent, and the sex ratio of pups is at parity (Helle 1980; Smith 1987). We used a fertility rate of 0.7 in our model, as calculated from data gathered from hunted seals (*N* = 579) in the Baltic (Helle 1977). Several landlocked seal populations show very high adult survival rates, perhaps partly caused by the lack of top predators such as polar bears (*Ursus marittimus*), sharks, and killer whales (*Orcinus orca*). Caspian seals and Baltic ringed seals often become more than 40 years of age (Gadjiev and Eybatov 1995). From this, we selected an adult survival rate of 0.95, which is close to the upper bound for other long-lived phocid seals such as the harbor seal and the gray seal (Härkönen and Heide-Jørgensen 1990; Harding et al. 2007). The age at first parturition varies among populations, but is generally around 6 years of age (Smith 1987; Reeves 1998). The maximum pup survival was set at 0.65 (Fedoseev 1975) and we iterated the last parameter value of subadult survival (0.89) to match the observed population growth rate (see “Results”). Limited combinations of survival values of young age classes are possible for a given growth rate due to life cycle constraints of seals (Härkönen et al. 2002; Harding et al. 2007).

Pup survival is linked to the annual ice conditions, and varies among years resulting in changes in the population growth rate (see below). Furthermore, we chose to use a fully age-structured model which accounts for effects from fluctuations arising from annual variations in pup survival, as single missing cohorts can affect the population growth rate during a number of years (Härkönen et al. 2002).

The population was projected forward 1 year at a time by multiplying the number of individuals in each age class with the life-history matrix:

4 $$ N_{{\left( {t + 1} \right)}} = A_{t} N_{t} , $$

where *N*_*t*_ is the age-structured population vector and *A*_*t*_ is the population projection matrix, which is modified at every time step *t*. Annual variations in pup survival (*p*_p(t)_) caused by temporal variations in availability of breeding ice is incorporated according to Eq. 5.

5 $$ A_{t} = \left[ {\begin{array}{*{20}c} 0 & 0 & f & \cdots & f \\ {p_{p\left( t \right)} } & 0 & 0 & \cdots & 0 \\ 0 & {p_{s} } & 0 & \cdots & 0 \\ \vdots & \vdots & \vdots & \ddots & \vdots \\ 0 & 0 & {p_{a} } & {} & 0 \\ \end{array} } \right] $$

The initial population sizes in each of the three regions were calculated from number of observed seals during aerial surveys (60 %): 10 040 seals in the Bothnian Bay (Fig. 3), 2500 in the Gulf of Riga, and 420 in the Gulf of Finland that amounts to a total Baltic population size of 12 960 (Härkönen et al. 1998; Misha Verevkin, Pers. Comm.). Initial stable stage structures were assumed in all three sub-populations. Estimated population sizes are mean sizes estimated from 1000 temperature trajectories. All simulations were carried out in MATLAB^®^.

### Linking Climate to the Demographic Model

Densities of birth lairs vary among ringed seal populations (Lydersen and Gjertz 1986; Lydersen et al. 1990; Smith and Lydersen 1991). Therefore, we parameterized the simulation model with a number of different biologically realistic territory sizes, ranging between 0.5 and 2.5 km^2^. We also estimated the extent of available breeding ice over the past 350 years based on historical ice coverage (Axell and Lindquist 2007), and run the population model with different territory sizes to find scenarios that could support the historical population size of 190 000 ringed seals (Harding and Harkonen 1999).

Competition for breeding ice is predicted to gradually affect the pup survival as the density of seals increases. We assume pup survival to be at maximum (0.65) when the amount of breeding ice is not limiting, and to decline linearly to zero as available breeding ice decreases. This is, by purpose, the only density-dependent effect incorporated in the model. We assume seals to be panmictic within regions, whereas no migrations occur among them, which are supported by data (Harkonen et al. 2008a). The life-history transition matrix (*A*_*t*_) was updated each year with the estimated pup survival taking annual population size and sea ice area into account. This generated time-varying matrices *A*_0_,…*A*_*T*_, representing each region spanning between time 0 (year 2010) and time *T* (year 2100). All calculations were repeated for 1000 temperature trajectories (realizations of the sequences of annual temperatures taken from the climate model), from which a mean population size and its standard deviation were calculated for each year and region.

## Results

Numbers of ringed seals hauled out during molt in the Bothnian Bay increased from about 2000 in 1988 to 6040 in 2011, which corresponds to an annual rate of increase of 4.6 % (Fig. 3). This growth rate is used in our simulations as described in “Materials and methods”.

**Fig. 4:**
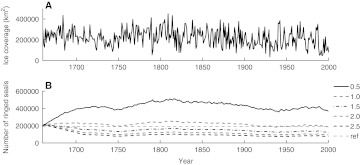
**a** Maximum annual ice extent in the Baltic Sea for the period 1660–2000 (Axell and Lindquist 2007). **b** Population trajectories for the Baltic ringed seal, as generated from the time series shown in **a**, where female territory sizes range between 0.5 and 2.5 km^2^. The *thin dashed line* indicates the pristine population size of 190 000 ringed seals in the Baltic Sea in the beginning of the twentieth century (Harding and Harkonen 1999)

Running the model with the estimated extents of breeding ice over the past 350 years we find that a territory size of 1.0 km^2^ permits a population size of about 200 000 ringed seals (Fig. 4), which is in the same order of magnitude as estimated from bounty statistics (Harding and Harkonen 1999). Consequently, we will focus on this territory size in the projections for future scenarios and also investigate possible effects of smaller and greater territory sizes.

**Fig. 5:**
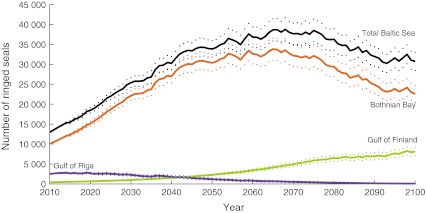
Mean and standard deviation of 1000 simulated scenarios of population growth for the Baltic ringed seal from 2010 to 2100. Projected population sizes for: the total Baltic Sea population, the Bothnian Bay sub-population, the Gulf of Finland sub-population, and the Gulf of Riga sub-population

The Bothnian Bay sub-population is projected to reach its maximum at 33 800 seals in 2065, and subsequently decrease to 22 550 in 2100 (Fig. 5). The ringed seal sub-population in the Gulf of Finland shows a different pattern with a continuous positive growth rate. This is explained by its currently small size (420 seals), which is why the amount of breeding ice per reproductive female is sufficient even during winters with low ice coverage. Detectable impacts on population growth can only be seen in the end of this century (Fig. 5). Projected numbers of seals in this area are 8100 in 2100. The Gulf of Riga sub-population, starting at 2500 seals, is predicted to reach its maximum at 2820 seals already in 2019, after which it decreases rapidly to only 75 in 2100 (Fig. 5). The total Baltic ringed seal population is projected to peak at 38 740, in 2068 and then decline to 30 730 in 2100.

Lack of breeding ice will result in hampered or declining growth rates in all sub-populations at the end of this century, but the Gulf of Riga sub-population will experience the most dramatic changes as winters get warmer and could become seriously threatened, especially since current number only amounts to 2500 seals.

When using territory sizes ranging from 0.5 to 2.5 km^2^ in the model, projected numbers of future population sizes differ, for instance the total population size at the end of the century would be 55 200 with a territory size of 0.5 km^2^ and 13 700 with a territory size of 2.5 km^2^. However, the main prediction of declining population sizes in Bothnian Bay and Gulf of Riga in the next century remains, regardless of chosen territory size.

As a reference point, if ice would not be limiting and the population would increase with 4.6 % a year, it would only take 60 years for the population to reach the numbers of 190 000, which is the estimated population size in the early twentieth century (Harding and Harkonen 1999). Including ice limitation, the population is projected to peak at 20 % of historical population size and then decline to 16 % at the end of the century. Consequently, ice limitation alone will severely hamper the recovery of the Baltic ringed seal population. Such effects were not found when the model was driven by historical ice conditions (Fig. 4).

## Discussion

Our model was constructed to scrutinize the effect of decreasing ice coverage on the population dynamics of Baltic ringed seals. Two main physical aspects affect the model outcome: future temperature and the correlation between temperature and ice quality. Seasonal mean temperatures predicted by the RCA3-model are generally within ±1 °C when compared to the observed data (Kjellström et al. 2005), adding confidence to this aspect of the study. Furthermore, correlations between air temperature and ice are reasonably well established (Meier et al. 2004).

The least known factor in this study is the relationship between pup survival and female territory size. We used a linear relationship between the amount of breeding ice available per adult female and pup survival, where the latter varied between 0 and 0.65. However, there could be a more abrupt threshold (minimum territory size required to nurse a pup successfully), after which pup survival drops dramatically. Females of small phocid seals such as harbor seals (*P. vitulina*) and ringed seals exhaust their energy reserves during lactation and forage particularly in the end of the nursing period (Härkönen and Heide-Jørgensen 1990; Hammill et al. 1991; Bowen et al. 2001). The occasionally common occurrence of starving ringed seal pups both in the Arctic (Smith 1987) and the Baltic (Olofsson 1933) indicate that some females cannot complete nursing successfully, resulting in “dwarf forms” of pups with high mortality rates (Olofsson 1933; Smith 1987).

Other climate-related factors than reduction of ice coverage may contribute to an even larger reduction in pup survival. Reduced snow depth could lead to lower recruitment as birth lairs become less stable (Ferguson et al. 2005). Climate models also suggest that the ice will break up earlier in the Baltic ringed seal breeding areas in the future (Meier et al. 2004), but this factor was not taken into account in the present model. Less ice could also lead to stronger competition between adults for territories and food also affecting adult survival (Freitas et al. 2008).

The risk for extirpation is high for the Gulf of Riga sub-population as Baltic ringed seals are stationary within the studied regions (Harkonen et al. 2008a). The series of warm winters with poor ice conditions after 1990 have already resulted in growth rates close to zero in the two southern regions, and in some years no pups survived due to early break up of ice (Mart Jüssi, Pers. Comm.).

We here focussed on the effects of warmer winters on Baltic ringed seals, but other well-documented threats to all landlocked seals include, organochlorine pollution causing impaired endochrinological functions and sterility (Helle 1980), infectious diseases such as phocine and canine distemper (Ohashi et al. 2001), over-fishing and signs of malnutrition (Karlsson and Bäcklin 2009). Thus, the effect of global warming we have investigated comes on top of a number of factors already severely affecting these endemic seal populations. Unless dedicated management actions are implemented, decreasing extent of ice could be the critical factor jeopardizing the future persistence of these seal populations.

Other landlocked seal species are likely to be affected in a similar manner as the Baltic ringed seal. The northern ice fields in the Caspian Sea form the critical breeding habitat of the endemic Caspian seal that give birth to their pups on the bare ice in February (Krylov 1990). Diminishing ice fields and reduction of the ice-covered period in the future will put additional pressure on a currently declining species (Harkonen et al. 2008b). Ladoga seals build their birth lairs in snowdrifts at the shoreline, and in pressure ridges that form in the central-northern part of the Lake Ladoga (Kunnasranta et al. 2001). Shorter and milder winters will reduce the offshore breeding habitat and increase the predation pressure by red foxes (*Vulpes vuples*), wolves (*Canis lupus*), and avian predators, as lairs are more likely to collapse under such conditions (Sipilä 2003; Ferguson et al. 2005). As Baikal seals build lairs close to the shore where snow accumulates (Ognev 1935), effects of changes in water level as expected from global warming can be expected to affect also seal species inhabiting the shores of Eurasian lakes.

## Conclusions

By linking a climatological model to a detailed population model, we have shown that the area of breeding ice as such imposes a strong regulating factor for the Baltic ringed seals. Even though breeding ice is the only density-dependent effect incorporated in the model, reduced growth rates are predicted in all three sub-populations in the coming century, and the southernmost sub-population will be most severely affected. The total Baltic ringed seal population, currently 12 960 seals, is predicted to amount to a mean of 30 730 seals in the year 2100, which is only 16 % of the population size in the beginning of the twentieth century (Harding and Harkonen 1999), and the population will thus remain small compared with earlier conditions. The risk for extirpation is high for the Gulf of Riga sub-population as the predicted mean amount to only 75 seals in the end of the century.
